# Effects of Age, Adipose Percent, and Reproduction on PCB Concentrations and Profiles in an Extreme Fasting North Pacific Marine Mammal

**DOI:** 10.1371/journal.pone.0096191

**Published:** 2014-04-22

**Authors:** Sarah H. Peterson, Jason L. Hassrick, Anne Lafontaine, Jean-Pierre Thomé, Daniel E. Crocker, Cathy Debier, Daniel P. Costa

**Affiliations:** 1 Ecology and Evolutionary Biology Department, University of California Santa Cruz, Santa Cruz, California, United States of America; 2 Laboratoire d’Ecologie Animale et Ecotoxicologie, Université de Liège, Liege, Belgium; 3 Department of Biology, Sonoma State University, Rohnert Park, California, United States of America; 4 Institut des Sciences de la Vie, Université catholique de Louvain, Louvain-la-Neuve, Belgium; Northwest Fisheries Science Center, NOAA Fisheries, United States of America

## Abstract

Persistent organic pollutants, including polychlorinated biphenyls (PCBs), are widely distributed and detectable far from anthropogenic sources. Northern elephant seals (*Mirounga angustirostris*) biannually travel thousands of kilometers to forage in coastal and open-ocean regions of the northeast Pacific Ocean and then return to land where they fast while breeding and molting. Our study examined potential effects of age, adipose percent, and the difference between the breeding and molting fasts on PCB concentrations and congener profiles in blubber and serum of northern elephant seal females. Between 2005 and 2007, we sampled blubber and blood from 58 seals before and after a foraging trip, which were then analyzed for PCBs. Age did not significantly affect total PCB concentrations; however, the proportion of PCB congeners with different numbers of chlorine atoms was significantly affected by age, especially in the outer blubber. Younger adult females had a significantly greater proportion of low-chlorinated PCBs (tri-, tetra-, and penta-CBs) than older females, with the opposite trend observed for hepta-CBs, indicating that an age-associated process such as parity (birth) may significantly affect congener profiles. The percent of adipose tissue had a significant relationship with inner blubber PCB concentrations, with the highest mean concentrations observed at the end of the molting fast. These results highlight the importance of sampling across the entire blubber layer when assessing contaminant levels in phocid seals and taking into account the adipose stores and reproductive status of an animal when conducting contaminant research.

## Introduction

Persistent organic pollutants such as polychlorinated biphenyls (PCBs) are harmful to wildlife because they can disrupt endocrine and immune function [1]–[3]. These effects are especially pronounced in top predators, including marine mammals, because persistent organic pollutants accumulate in adipose tissue and biomagnify with increasing trophic position [4]. The chemical and physical structure of PCBs allows these compounds to persist in the environment long after leaving human-derived sources and, as a result, they are detected in remote regions of the world [5], [6]. In general, worldwide, manufacture of PCBs ceased from 1972–1984, with the most recent PCB cessation occurring in Russia in 1993 [7]. PCBs are no longer uniformly increasing in the environment [6], [8], but their presence in marine and terrestrial ecosystems is widespread.

Demographic and physiological parameters such as age, sex, and adipose stores may affect persistent organic pollutant concentrations. Variability in concentrations within a population may cause certain individuals to be more at risk from the negative effects of persistent organic pollutants. The percent of adipose tissue often has a significant, negative correlation with contaminant concentrations, and fluctuations in adipose tissue as a result of prolonged fasting can increase contaminant concentrations in remaining tissues [9]–[15]. Persistent organic pollutant concentrations are often higher in males than females because females pass contaminants to offspring while males are unable to offload their contaminant burden [16]–[18]. For marine mammals, lack of depuration by males results in a positive relationship between age and contaminant concentrations, whereas reproductively active females do not demonstrate the same relationships between age and total contaminant concentrations. Insignificant age trends were observed for many reproductively aged female pinnipeds (seals, sea lions and the walrus), including harbor seals (*Phoca vitulina*) [17], Steller sea lions (*Eumetopias jubatus)* [16] and ringed seals (*Pusa hispida)* [18], suggesting that these species annually offload contaminants to their offspring and subsequently accumulate new contaminants while feeding over the remainder of the year. Conversely, adult female cetaceans, including fin whales (*Balaenoptera physalus*), pilot whales (*Globicephala melas*), Dalls porpoises (*Phocoenoides dalii*), and killer whales (*Orcinus orca*) had declining PCBs in blubber with increasing age [19]–[22], although that relationship may change after reproductive senescence [22], [23]. Both pinnipeds and cetaceans do not equally transfer all congeners to their offspring. Offspring are observed with a higher proportion of low-chlorinated congeners than their mothers [14], [24]–[27], which may affect congener profiles in adult females over time.

The major source of contaminants to marine mammals is from their food [4], [28]. Therefore, PCB concentrations in wildlife may depend on both foraging location and trophic position. Within similar trophic levels, previous studies showed foraging location to be important. Bottlenose dolphins (*Tursiops truncatus*) foraging adjacent to point-sources of PCBs showed higher concentrations than bottlenose dolphins foraging farther from point sources [29]. Mid-trophic level predators from the northeast Pacific Ocean, including albatrosses (*Phoebastria* spp.), humpback whales (*Megaptera novaeangliae*), killer whales, and harbor seals displayed geographically-associated variability in total PCB concentrations [22], [30]–[33]. The majority of animals from these studies were epipelagic (0–200 m depth) marine predators that also forage within the narrow margin of the continental shelf. Little is known about PCBs in mesopelagic (200–1000 m depth) open-ocean food webs and their effect on predators in these systems.

Assessing contaminants in free-ranging, open-ocean marine predators is complex because demographic parameters, estimates of adipose percent, and indices of foraging behavior are difficult to concurrently obtain from wild and visibly healthy marine predator populations. Northern elephant seals (*Mirounga angustirostris)* are unique in that all of these variables can be quantified when they arrive on land. They have been monitored at the Año Nuevo colony (California, USA) since 1968, where flipper tags are attached to several hundred pups annually, providing a consistent subset of known-age animals within the population that can be used to investigate relationships between age and contaminant concentrations. Northern elephant seals are long-lived, mesopelagic, high-trophic level predators. Based on diving behavior [34], [35], jaw-motion recorders paired with cameras [36], and stomach content analysis [37], northern elephant seals are hypothesized to consume mesopelagic fish and squid. However, little is conclusively known about their diet, since they return from their foraging migrations with nearly all prey completely digested.

Tracking data show that individual elephant seals from the Año Nuevo colony forage in distinct open-ocean regions, including the remote Pacific subarctic gyre [34], [35], [38], and near-coastal regions [34], [35], [39]. The northern elephant seal uses four hydrographic ecoregions within the northeastern Pacific, as defined by Longhurst [40], and is the only pinniped species in these ecoregions that consistently forages in the mesopelagic zone. Sampling a large number of individuals could capture the variability in contaminant concentrations between individuals that may utilize varying foraging strategies and obtain an appropriate range of contaminant concentrations for the population.

Elephant seals are also ideal for examining the relationships between adipose percent and contaminant concentrations during the extreme fasting periods they exhibit on land associated with breeding and molting. Many marine predators go through annual periods of fasting, often associated with long migrations, and the associated mass loss could significantly affect contaminant concentrations in different tissues. Elephant seals return to land from their biannual foraging migrations to breed and molt [34], [35]. While on land for breeding/lactation or molting, elephant seals cease feeding and go through extreme reductions in adipose stores, losing up to 40% of their body mass [41]. Previous studies of PCB concentrations in stranded northern elephant seals along the California coast [42], [43] demonstrated the capacity for PCBs in elephant seals to reach concentrations of toxicological concern [44]; however, we still do not know how concentrations fluctuate in healthy, wild animals throughout the year in relation to the naturally occurring extremes of adipose percent. The unique life history strategy of elephant seals, with separate fasting periods for breeding/lactation and molting, can allow us to disentangle the effects of fluctuating adipose percent with the effect of maternal offloading of contaminants. Elephant seal females begin reproducing between the ages of three and six [45]. Females have not been observed to go into reproductive senescence like many cetaceans and average natality is over 80% [35].

Our study builds on the findings of previous research [9], [13], [46] by incorporating adipose percent estimates for known-age adult females during the breeding and the molting fast. The main objectives of our study were to examine the potential effects of age, adipose percent, and reproductive state (breeding fast versus molting fast) on PCB concentrations and congener profiles in blubber and serum of northern elephant seal females. By investigating these parameters concurrently we hypothesized that 1) age would not affect total PCB concentrations but would affect congener profiles, resulting in older females with higher chlorinated congeners than younger seals (lower chlorinated congers may be more easily depurated to offspring), 2) total PCB concentrations would increase with decreased adipose percent, and 3) seals would have higher concentrations at the end of molting than at the end of breeding due to the lack of depuration to offspring during the molt.

## Methods

### Ethics Statement

The University of California, Santa Cruz Institutional Animal Care and Use Committee reviewed and approved the animal use protocols for this research. Research was carried out under National Marine Fisheries Service permit #87–1743.

### Animal Sampling

**Figure 1:**
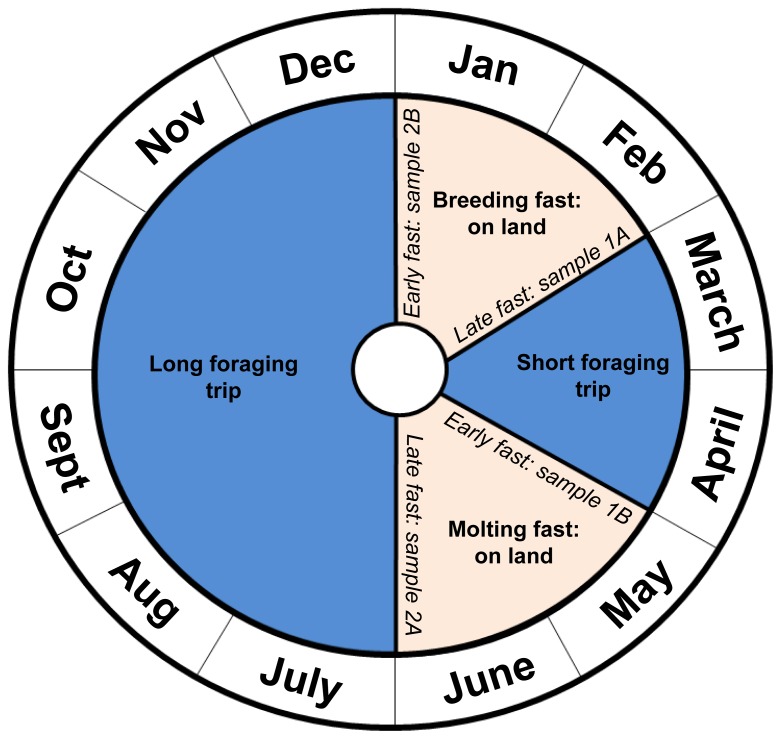
One year in the life of a female northern elephant seal. Tissue samples from the same animals (i.e. 1A/1B and 2A/2B) were collected just prior to the start of a foraging trip (late fasting) and just after the end of that foraging trip (early fasting).

Adult female northern elephant seals (N = 58) with no visual health impairments, were selected for tissue sampling at Año Nuevo State Reserve in San Mateo County, California, USA between 2005 and 2007. Most seals (N = 54) were known-age individuals between 4–17 years old, identified by a uniquely numbered, plastic tag (Dalton jumbo Roto-tags, Oxon, UK) placed in a lobe of their hind flipper. Female elephant seals have two distinct periods of fasting on land (breeding/lactation and molting) between foraging trips (Fig. 1). The annual breeding fast (January – February) is followed by a short foraging trip (February – April). This is followed by a molting fast (May – June), which precedes a long foraging trip (June – January). These foraging trips are hereafter referred to as the short and long trips. Individual seals were sampled for the first time at the end of one of the fasting periods (late breeding or late molting) just prior to departure to sea. Those seals that completed the foraging trip and returned to land were then sampled for a second time at the start of the next fasting period (Fig. 1). Not all seals returned from the foraging trip; therefore, pre-and post-foraging trip samples were paired for only some animals (Table 1). Seals were handled a maximum of two times during this study. Seals were chemically immobilized using standard protocols while morphometric measurements were taken and tissue samples (blubber and blood) were collected [34], [39]. Full thickness blubber cores were collected using sterile, single-use 6 mm biopsy punches (Miltex, Inc., York, Pennsylvania, USA) from the lateral pelvic area, wrapped in aluminum foil marked to identify inner and outer ends of the sample, stored on ice in the field, and frozen at −20°C until analysis [9]. Blood samples from the extradural vein were collected in 10 mL serum vacutainers and stored on ice in the field. At the laboratory, serum was separated by centrifugation and stored in cryovials at −20°C until analysis.

**Table 1:** ∑PCB concentrations in northern elephant seals (N = 58).

Tissue		Late breeding fast	Early molting fast	Paired	Late molting fast	Early breeding fast	Paired
		(Sample 1A)	(Sample 1B)	samples	(Sample 2A)	(Sample 2B)	samples
Inner	Sample size	N = 18 (17)	N = 13 (13)	13	N = 16 (12)	N = 31 (25)	8
	Mean ± sd	1241±545	884±179		1647±574	690±160	
	(range)	(495–2502)	(557–1204)		(708–2722)	(473–1120)	
Outer	Sample size	N = 19 (18)	N = 16 (15)	14	N = 18 (13)	N = 32 (25)	8
	Mean ± sd	912±262	1170±314		1003±336	900±221	
	(range)	(497–1461)	(777–2024)		(356–1597)	(504–1417)	
Serum	Sample size	N = 20 (19)	N = 16 (15)	14	N = 20 (15)	N = 29 (23)	8
	Mean ± sd	818±210	1207±517		1061±343	838±255	
	(range)	(542–1362)	(770–2837)		(723–1772)	(511–1495)	
Serum	Mean ± sd	7.8±1.8	9.0±3.1		8.1±2.6	6.2±1.4	
(wet weight)	(range)	(4.6–10.9)	(6.0–17.9)		(4.9–14.3)	(4.3–9.4)	

∑PCB concentrations are lipid-normalized (ng g^−1^ lipid) for inner blubber, outer blubber and serum. Serum ∑PCB concentrations are also reported by wet weight (µg L^−1^). Samples were collected at four different times of year (see Fig. 1) from 2005–2007.

Note. A subset of animals with known ages and body composition measurements were used for statistical analyses (sample sizes in parentheses). Late breeding (1A) – early molt (1B) samples were taken before and after the short foraging trip and late molt (2A) – early breeding (2B) samples were taken before and after the long trip. Numbers of paired samples (same seal) are given for each tissue type because not all seals were repeatedly sampled. Some seals were sampled twice while other seals were only sampled once.

All 58 adult females were included in our summary of PCB concentrations (Table 1). Only known age females with mass and estimates of adipose percent that reproduced normally were included in our statistical analyses (N = 50 seals). Not every seal had usable samples for all tissue types; therefore, subsets of the 50 seals were used to analyze PCB concentrations in inner blubber, outer blubber, and serum (Table 1). Paired late fasting (pre-foraging trip) and early fasting (post-foraging trip) samples of at least one tissue type (inner blubber, outer blubber or serum) were collected from 27 of the seals.

Girth and length measurements were taken at eight locations along the seal, including six locations where blubber thickness was measured dorsally, laterally and ventrally using a handheld ultrasound backfat meter (Scanoprobe, Ithaca, New York, USA) [35]. Body mass was determined by rolling the seal onto a canvas sling and using a hand-winch to suspend them from a 1000±1.0 kg Dyna-Link digital scale attached to a metal tripod [35]. The percent of adipose tissue was estimated using the morphometric measurements and a truncated cones technique [47], [48].

### Chemical Analyses

Blubber biopsies were cut into three equal parts. Inner (closest to the muscle) and outer (closer to the skin) blubber layers were analyzed in 2008 at the University of Liege. Using a Thermo Quest Trace 2000 gas chromatograph coupled with a ^63^Ni ECD (Thermo Quest, Trace 2000, Milan, Italy), serum and each section of blubber biopsy were analyzed separately for 22 PCB congeners (IUPAC 28, 44, 70, 87, 95, 101, 105, 110, 118, 128, 138, 149, 153, 156, 170, 180, 183, 187, 194, 195, 206, and 209). Details of sample preparation, clean-up, and analysis, including quality assurance, are provided in Debier et al. [49]. The limit of detection (LOD) was fixed at a level three times the background noise of the chromatogram. The limit of detection of PCB congeners was therefore 0.006 ng g^−1^ (ppb) of serum fresh weight, 2 ng g^−1^ (ppb) of serum lipid weight and 0.7 ng g^−1^ (ppb) of lipid weight of adipose tissue in our analytical conditions. The limit of quantification (LOQ) of PCB congeners was determined by means of spiked bovine serum and bovine fat samples used as quality control (QC) and was the lowest concentration which could be quantified and which showed a recovery range between 70% and 130% [50]. This concentration also corresponds to at least 10 times the background noise of the chromatogram. In these conditions, the LOQ for each PCB congener was 0.03 ng g^−1^ of serum fresh weight, 10 ng g^−1^ of serum lipid weight and 2.5 ng g^−1^ of lipid weight in adipose tissue. Congeners falling below the LOQ for elephant seal samples were recorded as measured concentrations. When a congener concentration was recorded as zero, this means that the congener was not detected at all in the sample. Blubber concentrations were quantified as ng g^−1^ lipid, while serum samples were quantified both by unit of serum lipid (ng g^−1^ lipid) and unit of whole serum (ug L^−1^). Lipids in serum were quantified as described in Vanden Berghe et al. [14]. In summary, different lipid classes (total cholesterol, phospholipids, triacylglycerols and nonesterified fatty acids) were quantified using enzymatic kits from Diasys (Diasys Diagnostics System, Holzheim, Germany) and Wako (Wako Chemicals USA Inc., Richmond, Virginia, USA). Concentrations of each lipid class were calculated using kit-specific recommendations, on the basis of standard equivalents. All lipid classes were added together to obtain serum total lipid concentrations.

### Statistical Analyses

#### Total PCB concentrations

Linear mixed effects (LME) models were run using the nlme package [51] in the statistical software R (version 2.15.2 [52]), to examine effects of age and percent adipose tissue (fixed effects) on total contaminant concentrations in inner blubber, outer blubber and serum. Models were run separately for each tissue type. Blubber composition is stratified and differences in fatty acid profiles have been observed between the blubber layers [53], [54]; therefore, our analysis focused on the inner and outer blubber layers. Models were run for both lipid-normalized serum ∑PCBs as well as ∑PCBs in serum measured per unit of wet weight. When models on serum ∑PCBs yielded the same trends, we only report lipid-normalized results. LME models included ∑PCB concentrations from samples collected at all time periods (Fig. 1). Residuals of each model were plotted against fixed effects to examine deviations from homogeneous variance, and data were log-transformed or appropriate variance structures were incorporated when necessary [55]. We treated individual as a random effect to account for repeated measurements for some individuals and the model was fit using restricted maximum likelihood (REML). For tests without repeated measures from individuals, general linear models were used to compare tissue concentrations at the end of the breeding and molting fasts, to examine potential influence of life history (i.e. fasting while lactating versus fasting with no offloading mechanism) on mean contaminant concentrations, while accounting for adipose percent.

The subset of individuals with paired pre- and post-foraging trip samples were additionally analyzed using paired t-tests to examine if ∑PCB concentrations in tissues (inner blubber, outer blubber, and serum) changed between the start and end of the short or the long foraging trip. T-tests were also used to examine whether the magnitude and direction of change in tissue concentrations were the same for both foraging trips (short versus long). The level of statistical significance for all analyses was set at p≤0.05.

#### PCB congener profiles

PCB concentrations were further examined at the level of specific congeners and congener families, based on the degree of chlorination. Low-chlorinated biphenyls included all congeners with three or four chlorines (tri- and tetra-CBs). The percentage of the total contaminant concentration made up by each congener family was calculated for every sample (inner blubber, outer blubber, and serum). Octa-, nona-, and deca-CBs were below the detection limit for the majority of samples, therefore statistical analyses were focused on tri/tetra-CBs, penta-CBs, hexa-CBs, and hepta-CBs. LME models were run with the percentage of each congener family as the response variable, after an arcsin square root transformation was applied [56], to examine the proportional contribution of each congener family to the total PCB concentration. Age and adipose percent were fixed effects and individual was included as a random effect.

## Results

**Figure 2:**
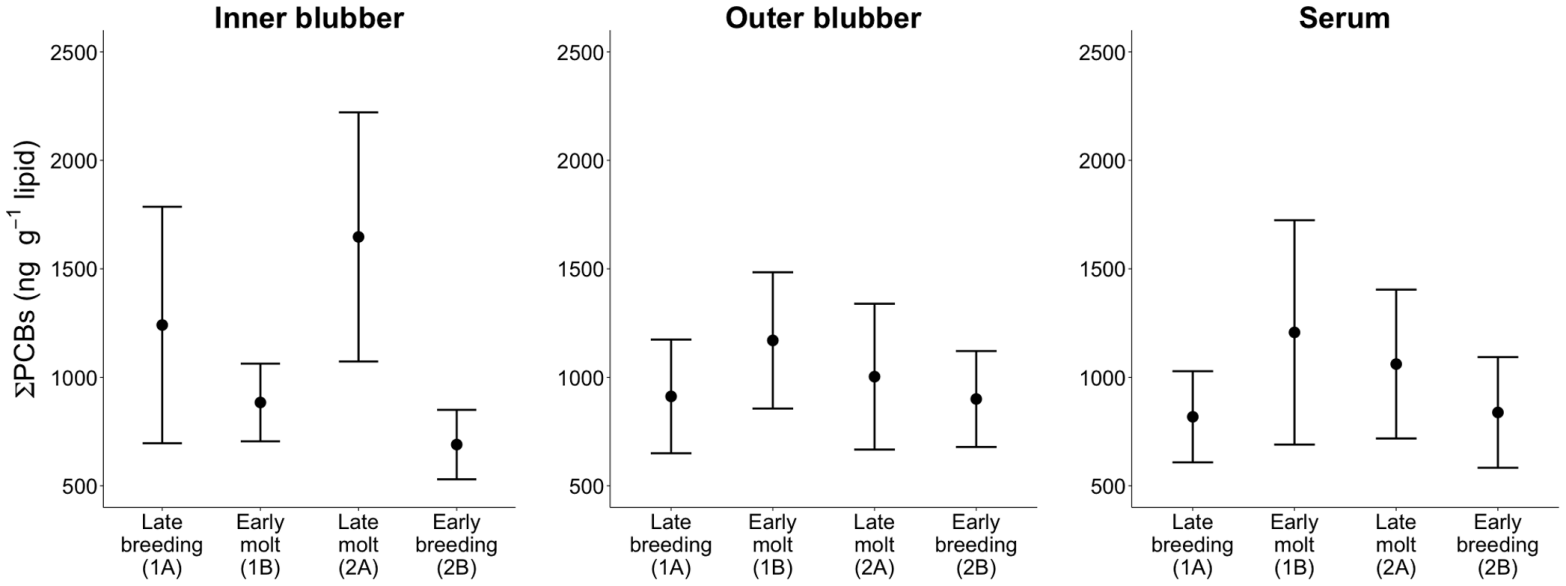
∑PCB concentrations (mean ± SD) for inner blubber, outer blubber, and serum samples measured in elephant seals during four different sampling periods (refer to Fig. 1 for timing of samples).

PCBs were detected in all samples collected from 58 adult female northern elephant seals, ranging from 4–17 years of age (Table 1). ∑PCB concentrations ranged from 356–2722 ng g^−1^ lipid in blubber and 512–2,837 ng g^−1^ lipid in serum (Table 1; Fig. 2).

**Figure 3:**
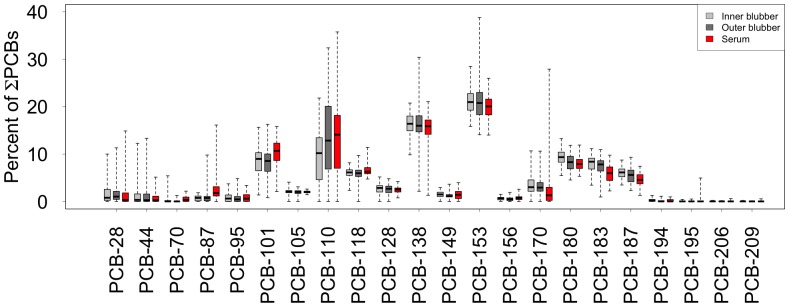
Proportions of each congener within the inner blubber, outer blubber, and serum of northern elephant seal females. Whiskers in these standard boxplots encompass the full range of the data. All sampling periods are combined, which means that some females contributed two samples while other females only are represented by one sample.

The majority of inner and outer blubber PCB profiles in female seals were comprised of eight congeners: PCB-101, -110, -118 (penta-CBs), -138, -153 (hexa-CBs), -180, -183, and -187 (hepta-CBs), all of which had a mean percent contribution of the total burden greater than 5% (Fig. 3). These eight congeners comprised 84.9±4.5% of the total PCB concentration in inner blubber and 90.2±3.6% in outer blubber. PCB-153 was the most common congener, comprising 15.8–28.5% and 14.1–38.8% of the ∑PCB burden in inner and outer blubber, respectively. Hexa-CBs were the dominant congeners in blubber for all sampling periods, on average comprising >40% of ∑PCBs. Octa-, nona-, and deca-chlorobiphenyls combined comprised less than 1.7% of the total blubber PCB burden, and octa-CBs were the most prevalent of these high-chlorinated congener groups.

Serum PCB profiles had seven congeners that contributed a mean percent of more than 5% of the total PCB concentration: PCB-101, -110, -118, -138, -153, -180, and -183. These seven congeners comprised 78.8±5.9% of the total serum concentrations. PCB-153 comprised between 14.0–26.4% of the total serum PCB concentrations.

### Influence of Age

**Figure 4:**
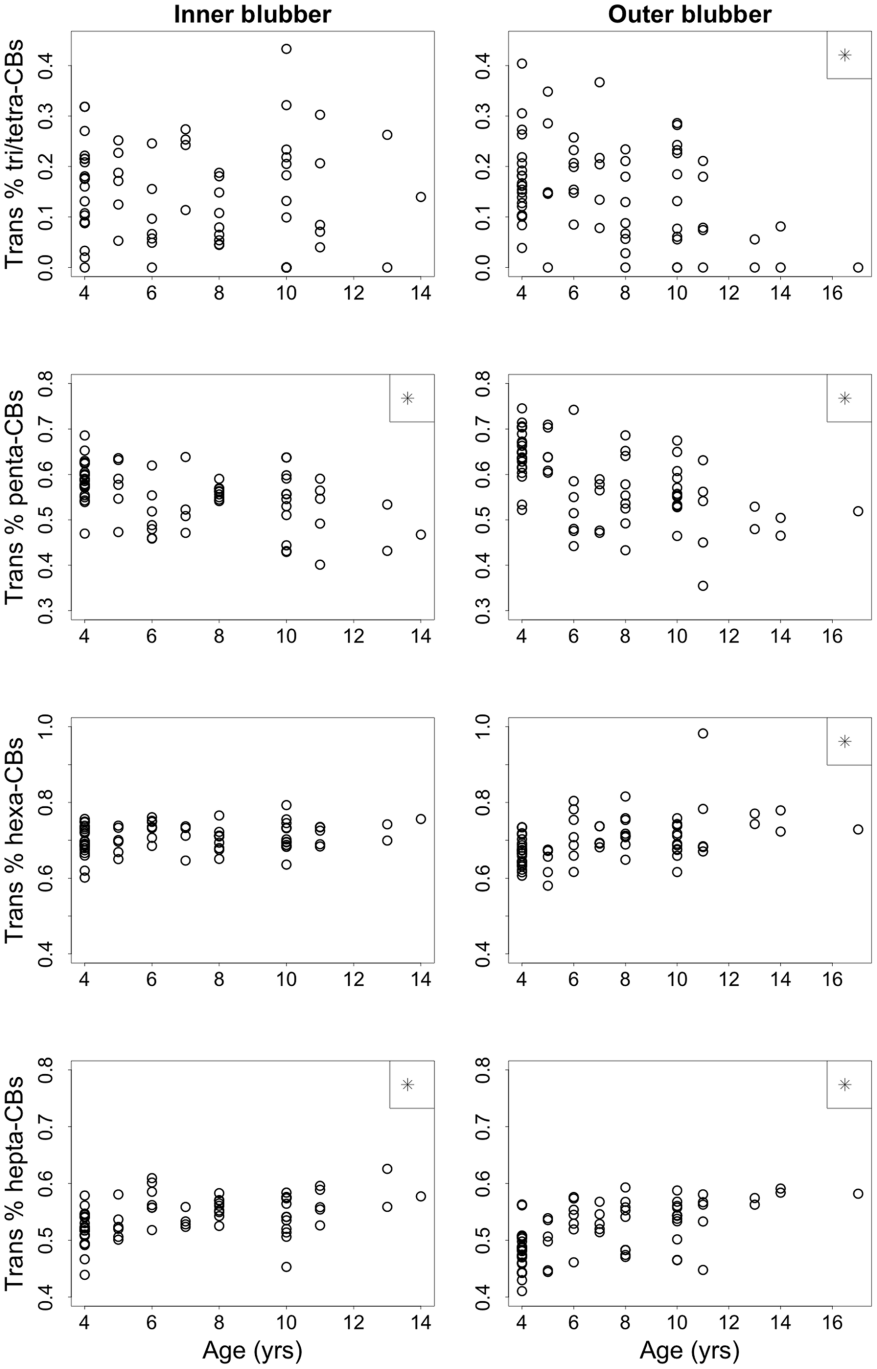
Inner and outer blubber congener profiles by age for all sampling periods combined. Statistics were run on transformed percentages (arc-sin square root). Asterisks indicate a stastically significant relationship with age.

There was no detectable relationship between age and total PCB concentrations in inner blubber, outer blubber or serum (p≥0.230). However, age had a significant effect on congener groups (Fig. 4; Table 2). There was a significant, negative relationship between age and the percent of penta-CBs (F_1,44_ = 12.8, p<0.001) and a significant, positive relationship between age and the percent of hepta-CBs (F_1,44_ = 12.4, p = 0.001) in inner blubber. There was no detectable relationship between age and the percent of either tri/tetra-CBs or hexa-CBs in the inner blubber. In the outer blubber, there was also a significant, negative relationship between age and the percent of penta-CBs (F_1,47_ = 30.1, p<0.001) and a significant positive relationship between age and the percent of hepta-CBs (F_1,47_ = 35.7, p<0.001). Additionally, in the outer blubber, the percent of tri/tetra-CBs had a significant, negative relationship with age (F_1,47_ = 15.1, p<0.001) and the percent of hexa-CBs had a significant, positive relationship with age (F_1,47_ = 15.8, p<0.001). The same significant, negative relationship observed for both blubber layers between age and the percent of penta-CBs was also detectable for lipid-normalized serum (F_1,47_ = 14.0, p<0.001) and serum concentrations using wet-weight. The percent of tri/tetra-CBs also had a significant, positive relationship with age in the serum (F_1,47_ = 7.2, p = 0.010), which was the opposite trend as observed in the outer blubber layer. The percent of hexa-CBs and hepta-CBs in serum did not change with age.

**Table 2:** Relationship between the percent of the ΣPCB concentrations in inner blubber, outer blubber and serum made up by each congener group and A) adipose percent or B) age.

Statistical test	Chlorination	Inner blubber	Outer blubber	Serum
Age	Tri/Tetra-CBs (3–4)	n.s.	–	+
	Penta-CBs (5)	–	–	–
	Hexa-CBs (6)	n.s.	+	n.s.
	Hepta-CBs (7)	+	+	n.s.
Adipose percent	Tri/Tetra-CBs (3–4)	+	+	n.s.
	Penta-CBs (5)	–	–	+
	Hexa-CBs (6)	n.s.	n.s.	n.s.
	Hepta-CBs (7)	n.s.	–	n.s.

Relationship indicated by statistically significant and positive relationship (+), significant and negative relationship (–), or no significant relationship (n.s.).

### Influence of Adipose Percent and Fasting State

Elephant seals were fattest early in the breeding season fast (mean ± SD: 34.7±2.3%; range: 30.9–39.9%) and leanest late in the breeding fast (mean ± SD: 29.3±1.7%; range: 26.0–32.0%). Seals sampled early in the molting fast had a mean adipose percent of 30.8±2.7% (range: 23.4–33.8%) compared to 30.9±2.4% (range: 26.1–36.1%) late in the molting fast, indicating that adipose and lean tissue were lost in similar proportions over the course of the molt.

**Figure 5:**
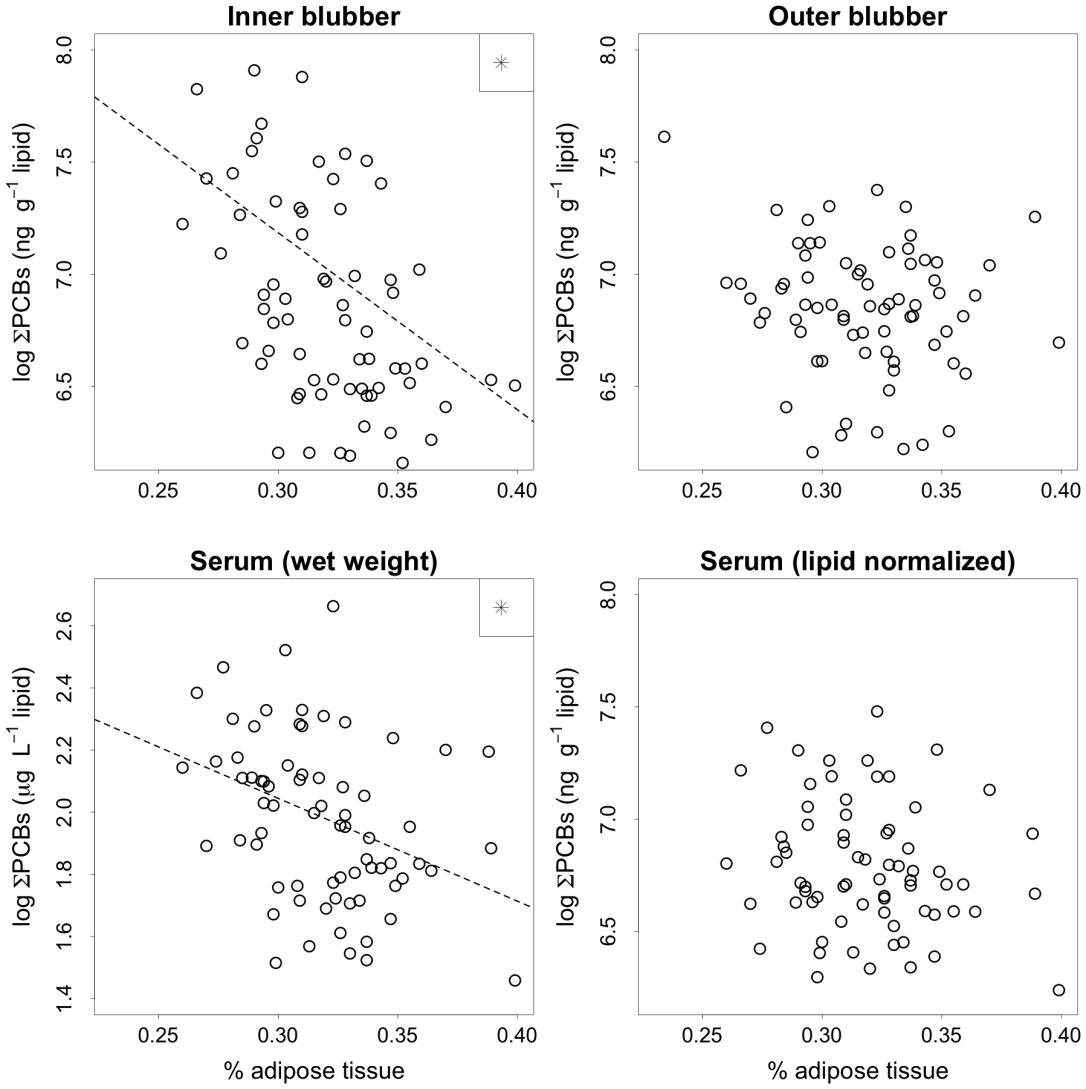
Inner blubber, outer blubber and serum (wet weight and lipid-normalized) ∑PCB concentrations relative to body condition (percent of adipose tissue). Statistical analyses were run using log transformed ∑PCB concentrations. Samples are from all sampling periods combined. Asterisks indicate stastically significant relationships.

**Figure 6:**
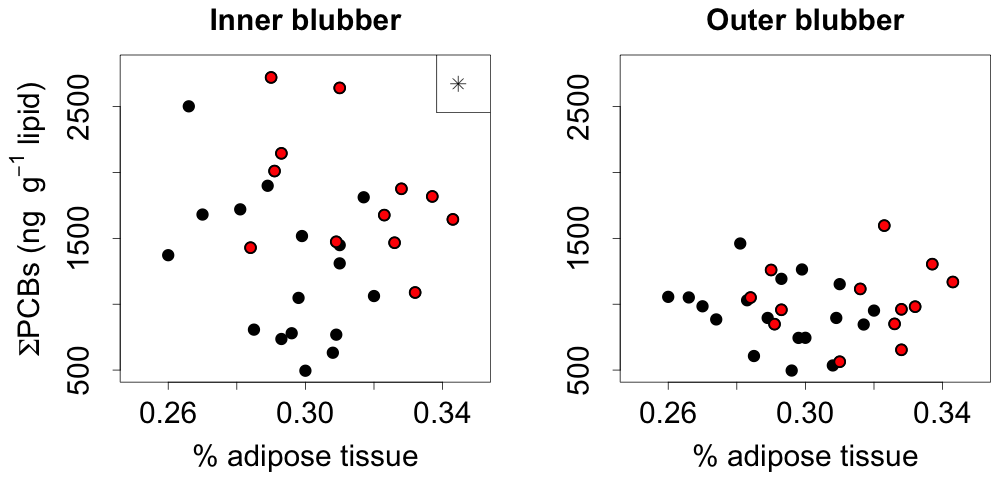
∑PCB concentrations in individual blubber samples at the end of the breeding/lactation fast (black) and the molting fast (red), relative to body condition (percent of adipose tissue). Asterisk indicates a stastically significant difference between groups.

The percent of adipose tissue had a significant relationship with total PCB concentrations, although this relationship was only observed for the inner blubber layer and serum measured by unit of wet weight and not for the outer blubber or lipid-normalized serum concentrations (Fig. 5). Overall, inner blubber ∑PCB concentrations were higher for seals that were leaner (F_1,20_ = 19.7, p<0.001). Direct comparisons of late breeding and late molting, while accounting for adipose percent, revealed that seals had higher mean inner blubber ∑PCB concentrations at the end of the molt (F_1,26_ = 13.1, p = 0.001) (Fig. 6). Conversely, outer blubber ∑PCB concentrations did not have a significant relationship with adipose percent (F_1,21_ = 2.4, p = 0.136), and there was no detectable difference between ∑PCB concentrations in late breeding and late molting outer blubber samples (F_1,28_ = 1.1, p = 0.310; Fig. 6).

The relationship between the percent of adipose tissue and total serum ∑PCB concentrations was dependent on whether the concentrations were analyzed using lipid-normalized or wet weight concentrations. Serum ∑PCBs measured per unit lipid initially appeared to have a significant relationship with adipose percent (F_1,21_ = 6.1, p = 0.023); however, when the single highest serum concentration was removed from analysis (2837 ng g^−1^ lipid measured during the early molting fast) this relationship became non-significant (F_1,20_ = 2.2, p = 0.157). Serum ∑PCB concentrations measured per unit of wet weight had a significant relationship with adipose percent even with the highest concentration removed (F_1,20_ = 10.1, p = 0.005). There was a significant difference between mean late breeding and late molting serum ∑PCB concentrations per unit lipid (F_1,31_ = 11.9, p = 0.002) but there was no significant difference between the late breeding and late molting serum ∑PCB concentrations per unit of wet weight (F_1,31_ = 1.6, p = 0.213).

Adipose percent had significant relationships with some congener groups in blubber and serum (Table 2). Fatter seals had a significantly greater percent of tri/tetra-CBs in their inner blubber than seals with lower adipose percentages (F_1,19_ = 13.6, p = 0.002), while the opposite was true for adipose percent and the proportion of penta-CBs in the inner blubber (F_1,19_ = 8.3, p = 0.010). There was no detectable relationship between adipose percent and the proportion of hexa-CBs or hepta-CBs in the inner blubber. In the outer blubber, adipose percent also had a significant, positive relationship with the proportion of tri/tetra-CBs (F_1,21_ = 12.9, p = 0.002) and a significant, negative relationship with the proportion of penta-CBs (F_1,21_ = 12.6, p = 0.002). Additionally, adipose percent had a significant, negative relationship with the proportion of hepta-CBs in the outer blubber (F_1,21_ = 18.8, p<0.001). There was no detectable relationship between adipose percent and the proportion of hexa-CBs in the outer blubber. In serum, there was a significant, positive relationship between adipose percent and the proportion of penta-CBs (F_1,21_ = 6.4, p = 0.020), the opposite trend to that observed in blubber. There were no significant relationships detected between adipose percent and the proportion of tri/tetra-CBs, hexa-CBs or hepta-CBs in serum.

### Paired Tissue Samples

**Figure 7:**
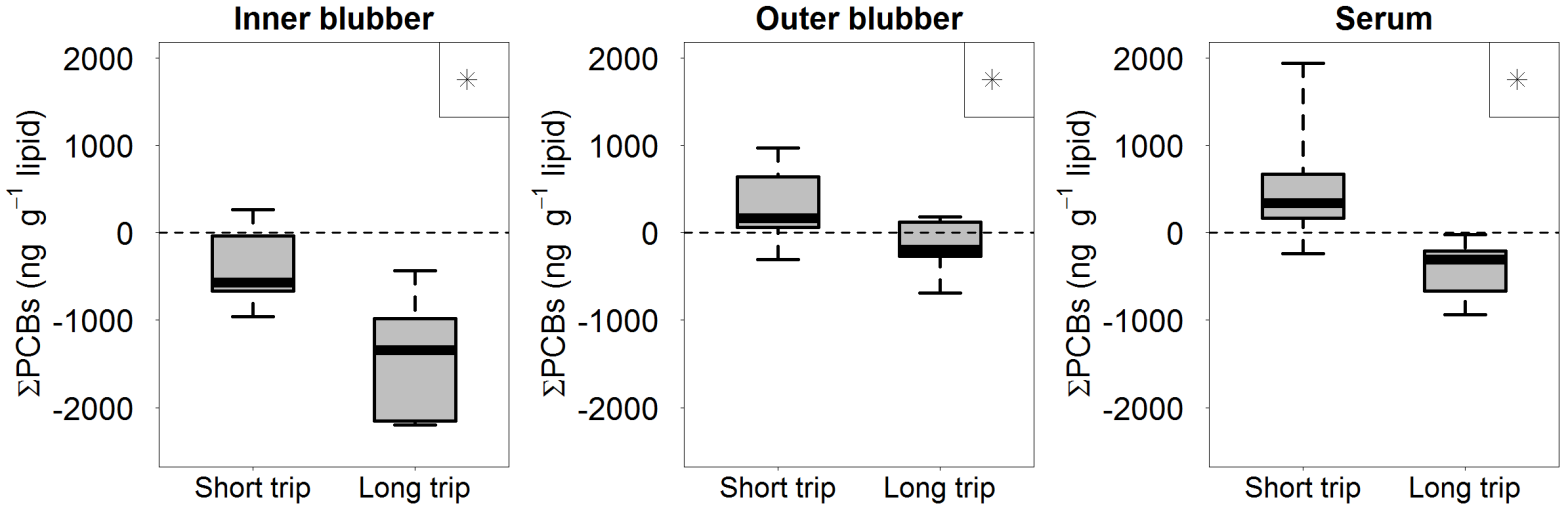
Changes in paired ∑PCB concentrations over the course of a foraging trip (same individuals pre- and post-foraging) for inner blubber, outer blubber and serum. Asterisks indicate statistically significant differences between the two trips. Refer to Fig. 1 for annual life history phases and differences between the short and long trip.

Paired tissue samples from the same individuals pre- and post-foraging revealed that changes in ∑PCB concentrations within individuals differed by tissue type and whether the change was measured across the short or long foraging trip (Fig. 7). The concentration of ∑PCBs in inner blubber decreased across the long (t = 6.2, df = 7, p<0.001) and short trips (t = 3.3, df = 12, p = 0.007); however, the magnitude of decrease was greater over the course of the long trip (t = 4.5, df = 19, p<0.001). The outer blubber layer changed in concentration across both the short (t = −2.8, df = 13, p = 0.015) and long trips (t = 2.3, df = 7, p = 0.05) but the direction of the change differed between the trips (t = 3.4, df = 20, p = 0.003). ∑PCBs increased in outer blubber over the course of the short trip but decreased over the course of the long trip. Lipid-normalized serum ∑PCB concentrations demonstrated the same differences between foraging trips as observed in outer blubber (t = 4.6, df = 20, p<0.001). Lipid-normalized serum ∑PCB concentrations increased over the course of the short trip (t = −3.1, df = 13, p = 0.008) but decreased over the course of the long trip (t = 3.7, df = 7, p-value = 0.008). Changes in ∑PCB concentrations in serum measured by unit wet weight did not significantly change across the short trip (t = −1.9, df = 13, p = 0.081) but significantly decreased across the long trip (t = 3.5, df = 7, p = 0.010), and changes in ∑PCB concentrations between the trips was significantly different (t = 4.2, df = 20, p<0.001).

## Discussion

Our findings highlight that toxic contaminants are detectible in seals that forage in remote depths of the Pacific Ocean, thousands of kilometers from anthropogenic sources. Mesopelagic and open-ocean foraging behaviors do not leave northern elephant seals immune to the accumulation of PCBs, as PCBs were detected in blubber and blood of all 58 sampled northern elephant seal females. ∑PCB concentrations in elephant seal serum and blubber sampled during early and late breeding in the present study are similar to those previously observed in elephant seal females [13] and weaned pups [9], [46] from the same breeding colony (Año Nuevo). However, the mean ∑PCB concentration in samples collected at the end of the molting fast in our study are higher than the mean ∑PCB concentration in samples previously collected at the end of the breeding fast [13]. Our study is the first to quantify ∑PCB concentrations in free-ranging, female northern elephant seals across the full range of naturally occurring body conditions and annual life history phases. Our results demonstrate the importance of accounting for both adipose percent and reproductive state when interpreting contaminant concentrations.

Quantification of ∑PCBs in marine mammals varies somewhat based on the number of congeners that are quantified in each study, which makes direct comparison of ∑PCBs or the percent of individual congeners difficult between studies. Additionally, the reporting of individual congeners varies between study, making one to one comparisons difficult. Nevertheless, some broad comparisons can be made between our observations and other published studies, realizing that the absolute values may differ slightly based on study design. Female northern elephant seals have substantially lower ∑PCB concentrations than stranded adult California sea lions (*Zalophus californianus*) [57], but adipose percentages are lower in most stranded animals than elephant seals from the present study. Blubber ∑PCB concentrations in elephant seals from our study are higher than those found in free-ranging, coastally-foraging adult female harbor seals from Kodiak Island, the southern Alaska Peninsula, and Prince William Sound [17], although these harbor seals forage further north than elephant seals from the present study and there are no comparable studies for free-ranging adult harbor seals foraging along the west coast of the United States of America. Given that all seals from our study harbor PCBs, greater sampling of animals across this region is warranted, especially because of the paucity of studies reporting ∑PCB concentrations for free-ranging adult pinnipeds from the eastern North Pacific. This would provide more complete documentation of baseline contaminant ranges within multiple species and the ability to appropriately compare species that utilize more similar geographic ranges.

The most common PCB congener quantified in elephant seal blubber for our study is CB-153, which contributes the greatest proportion to ∑PCBs in a broad range of marine mammal species [14], [17], [26], [58], [59]. The mean contribution of CB-138 and CB-180 in the present study places those congeners among the top five congeners contributing to the ∑PCB concentration in northern elephant seals. These same congeners are among the top five most important congeners for other pinnipeds, including adult fur seals (*Callorhinus ursinus*) [58], harbor seals [17] and Hawaiian monk seals (*Monachus schauinslandi*) [59] from the Pacific, as well as for gray seals [14] and harbor seals [26] from the Atlantic.

### Influence of Age

The absence of a significant age relationship with ∑PCB concentrations indicates that elephant seals likely offload contaminants during lactation and replenish contaminants while foraging. Age does not affect ∑PCB concentrations in the blubber of other female pinnipeds, including harbor seals [17], Steller sea lions [16], and ringed seals [18]. ∑PCB concentrations in female odontocete and mysticete blubber decrease with increasing age [19], [20], although this relationship reverses in post-reproductive females after reproduction senescence and the associated cessation of lactation [22], [23]. These observations indicate that there are fundamental differences in how contaminants accumulate in cetaceans and pinnipeds, possibly driven by life history strategies and reproductive senescence.

Based on previous research on elephant seals from Año Nuevo quantifying average ∑PCB concentrations in milk [13] and average milk production in female elephant seals [41], females may annually offload approximately 23.9 mg of PCBs during an average 26.5 day [41] lactation period. This estimated transfer for northern elephant seals is similar to the calculated transfer of PCBs (27.0±11.2 mg) from grey seal female to their pups [60]. Concentrations of ∑PCBs in adult female elephant seal blubber, serum and milk tissues (present study; [13]) are lower than the concentrations observed in some highly contaminated marine mammal species [22], [29], [31]. However, transfer of contaminants to the pup is during a critical period of pup development for the immune, endocrine, and nervous systems [24] and maternal transfer of PCBs can affect fetal brain development, as observed in rats [61]. It is unlikely that PCBs are the only anthropogenic contaminant acquired by an individual [13]; therefore, each seal could have additional contaminants within blubber or other tissue compartments. It is possible that contaminants may interact, potentially in a synergistic way, but this has yet to be fully examined.

Age significantly affects the proportion of PCB congener families in female elephant seal blubber and serum. This suggests a positive relationship between an age-associated process, such as the number of times a female has given birth, and the proportion of higher chlorinated congeners in elephant seal tissues. Congeners with increased chlorination are more lipophilic and have a higher octanol-water partitioning coefficient (K_ow_). This decreases the transfer efficiency from maternal blubber to offspring through milk, potentially due to differences of mobilization efficiency from blubber [14] as well as diffusion across the boundary of the mammary gland [14], [25], [62]. Congeners with higher log K_ow_ comprised a greater proportion of PCBs in maternal harbor seals [24], grey seals [14], [25], harbor porpoises [26], and belugas [27] than their offspring, which may occur in northern elephant seals and cause the relationships observed between age and specific congener families. Older female seals have increased parity and greater adipose mass loss and milk production during lactation, which leads to more opportunities to depurate congeners with lower K_ow_ values, while selectively retaining congeners with higher K_ow_ values. Another potential mechanism that can explain the age relationship observed in our study is the preferential metabolism of lower chlorinated congeners. It is possible that both selective excretion through milk and preferential metabolism could play a role in the age and congener trends observed in female northern elephant seals.

### Influence of Adipose Percent and Fasting State

Total PCB concentrations in inner blubber and serum (wet weight) have significant, negative relationships with the percent of adipose tissue. However, this trend is not observed in outer blubber or serum measured per unit lipid. This suggests that varying physiological and temporal mechanisms mediate fluctuations within the main reservoir (blubber) and the transport system (serum) of PCBs in northern elephant seal females. Asynchronous fluctuations between inner blubber, outer blubber, and serum are likely due to differential use of these tissue components during fasting and lactation, and the potential for transfer of contaminants to offspring. Trends observed here are supported by previous studies. In general, inner blubber ∑PCB concentrations may be more dynamic than outer blubber ∑PCB concentrations because inner blubber is the main energy reserve mobilized during fasting [63]. Fatty acid profiles for the inner blubber layer of adult female elephant seals change significantly over the lactation-associated fasting period, whereas the proportion of different classes of fatty acids in outer blubber remains stable through lactation [53]. Fatty acid profiles of inner blubber are similar to those observed in milk, which supports inner blubber as the main contributor of fatty acids to milk synthesis [53]. Additionally, outer blubber has a high proportion of medium chain length monounsaturated fatty acids, which are theorized to serve in a thermoregulatory capacity [53]. Thus, changes in ∑PCB concentrations correspond most strongly with changes in maternal lipid content, especially in the inner blubber layer.

When animals are at their fattest, outer blubber concentrations may be higher than inner blubber concentrations [13], [49]. ∑PCB concentrations may increase in the inner blubber while fasting, because an animal utilizes the blubber reserve from the inside first and concentrates contaminants in the remaining tissue. This may cause the inner blubber ∑PCB concentrations to become higher than the outer blubber ∑PCB concentrations by the end of fasting [13], [49], [53]. In the present study, we observe the same trend occurring during the molting fast, although the overall concentrations in inner blubber are highest at the end of the molting fast. This may be attributed to the life history phase, with seals operating more as a closed system during the molting fast because lactation does not occur during the molt and there is no offloading of contaminants to offspring (Table 1).

Biannual fasting periods of seals on land are associated with breeding or molting and result in significant reductions in adipose tissue and body mass [41], [64]. Female seals are on land for different life history stages (breeding/lactation and molting), which likely causes the differences in how energy stores are used during the two fasting periods. Female seals during the breeding season lose approximately 58% of their fat mass and lose significantly more fat than lean tissue [41]. Female seals during the molt lose an average of 38% of their fat mass and similar proportions of fat and lean tissue [64]. It is important to note that seals are at their fattest at the beginning of the breeding season and adipose reserves are not as great when seals arrive for the start of the molt, as seals have approximately three times longer to forage at sea prior to the breeding season than in between the breeding and molting seasons (Fig. 1) [35]. Accounting for fat stores, we observed that the highest mean ∑PCB concentrations in inner blubber occurred at the end of the molting fast, when seals were both relatively thin and unable to depurate contaminants to their offspring, rather than at the end of the breeding fast when seals were at their leanest. This suggests that both the fat content of the animal and its reproductive condition affect contaminant concentrations. Elephant seals only transfer contaminants to offspring during the long foraging trip (gestation) and the subsequent fasting period on land (lactation) and not during the molting fast; therefore, reproductive transfer to offspring, through the placenta and highly lipid-rich milk, may be responsible for this observed difference.

While concentrations of ∑PCBs in blubber and serum in our study are below the concentration threshold that corresponds with immune system suppression in harbor seals [44], concentrations in elephant seals fluctuate significantly relative to body condition. Elevated ∑PCB concentrations in inner blubber and the mobilization of PCBs to serum at the end of a fasting period, as observed in elephant seals [13] and grey seals [14], [49], could increase the vulnerability of individuals to cumulative effects from additional contaminants or physiological stressors that reduce their adipose tissue reserves and concentrate contaminants in remaining tissues. Our observations of increased ∑PCB concentrations at lower adipose percentages are consistent with observations in other marine mammals, including humpback whales and polar bears (*Ursus maritimus*), that undergo significant fasting periods [65], [66].

Animals may be especially vulnerable to the effects of contaminants if normal fasting causes increased tissue concentrations and is then followed by decreased post-fasting foraging opportunities. This was the possible explanation for elevated ∑PCB concentrations observed in a female from the present study. This female lost adipose tissue over the course of a short, post-breeding foraging trip, which corresponded to an increase in blubber and serum ∑PCB concentrations of 92% and 213%, respectively, over her late fasting concentrations. Her tissue concentrations, measured before and after the foraging trip, are among the highest observed during any time period and, based on our observations of other seals in this study, it is likely that her ∑PCB tissue concentrations would have been even higher at the end of the molting fast. Similar trends are observed in polar bears, where fluctuations in seasonal food availability outside of the hibernation period can influence body condition and thus affect PCB concentrations [66]. These observations have implications for other fasting animals, especially marine predators that fast while migrating or provisioning offspring.

### Paired Samples

Examining paired tissue samples from the same individuals before and after a foraging trip provides insight into certain dynamics that are not observable in a larger population level analysis. These results are unique because tissue samples were paired between the start and end of a foraging period and not between the start and end of a fasting period, as is common for studies on the PCB dynamics in the blubber and blood of marine mammals. Inner blubber is the most metabolically active layer in northern elephant seals [53] and ∑PCB concentrations in our study vary with adipose percent both at population-level and individual-level analyses. Individuals show a decrease in ∑PCB concentrations within the inner blubber layer over the course of a foraging trip; however, the magnitude of the decrease is greater over the long trip than the short trip, which may partially be explained by differences in adipose tissue gained over these trips [35] due to differences in trip length (Fig. 1). The ∑PCBs in outer blubber and serum at the individual level present a more complicated story, increasing in concentration across the short trip but decreasing in concentration across the long trip, which suggests that PCB dynamics in outer blubber may be more complex than previously thought.

### Conclusion

Overall, our findings demonstrate that PCBs accumulate in female northern elephant seals that forage across the mesopelagic north Pacific. While total accumulation of PCBs does not change with age, our findings reveal that more chlorinated PCB congeners comprise a higher proportion of the total PCB burden of older females while younger seals have a higher proportion of less-chlorinated PCBs. At this time, the health consequences of these observations remain unknown.

Northern elephant seals exhibit a set of life history strategies that depend on two seasonal fasting periods. Our findings suggest that female northern elephant seals are most vulnerable to high ∑PCB concentrations at low adipose percentages, which occur at the end of their two seasonal fasts. Depuration to pups may mitigate high blubber ∑PCB concentrations in adult female tissues during the breeding fast and prevent overall ∑PCB concentrations from increasing with increasing age. However, no mitigating mechanism is known to occur during the molting fast, and therefore, our results suggest the female elephant seals are most vulnerable to increased concentrations of ∑PCBs during their molting fast. Free ranging adult male elephant seals may accumulate significantly more ∑PCBs than females since they have no ability to mitigate their contaminant load through reproductive transfer. More research is needed on males to verify this hypothesis.

In addition, the inverse relationship between body condition and total PCB concentration in the inner blubber has implications for the development of bio-monitoring strategies. First, the presence of a significant, negative relationship between adipose percent and ∑PCB concentration in the inner blubber, but not in the outer blubber, indicates the complexity of blubber as a biological indicator. For elephant seals, our results suggest that ∑PCB concentrations of inner blubber represent recent trends in contaminant fluctuation, while ∑PCB concentrations of outer blubber, a tissue of slower turnover, may represent longer-term accumulation. Bio-monitoring of more elusive marine predators, where biopsy darting is the only option, may provide misleading results if only the outer-most tissue is sampled. It is imperative that adipose percent and the life history of an animal be fully considered for both the design of contaminant monitoring programs and appropriate interpretation of the results.
